# Vascular Complications of Long COVID—From Endothelial Dysfunction to Systemic Thrombosis: A Systematic Review

**DOI:** 10.3390/ijms27010433

**Published:** 2025-12-31

**Authors:** Maja Stojanovic, Marko Djuric, Irina Nenadic, Suzana Bojic, Ana Andrijevic, Aleksa Popovic, Slobodan Pesic

**Affiliations:** 1 Clinic for Anesthesiology and Intensive Care, Institute for Cardiovascular Disease, 11030 Belgrade, Serbia; ana.andrijevic@yahoo.com; 2 Faculty of Medicine, University of Belgrade, 11000 Belgrade, Serbia; drdjuric89@hotmail.com (M.D.); nenadicirina@gmail.com (I.N.); subojic@yahoo.com (S.B.); 3 Clinic for Anesthesiology and Intensive Care, University Clinical Hospital Center “Dr Dragisa Misovic-Dedinje”, 11030 Belgrade, Serbia; 4 Clinic for Vascular Surgery, Institute for Cardiovascular Disease, 11030 Belgrade, Serbia; aleksapopovic98@gmail.com (A.P.); spesic90@gmail.com (S.P.)

**Keywords:** COVID-19, Long COVID, systemic inflammation, vascular dysfunction, antiphospholipid antibodies, cytokine storm, endothelial dysfunction, coagulopathy

## Abstract

Coronavirus disease 2019 (COVID-19), caused by the severe acute respiratory syndrome coronavirus 2 (SARS-CoV-2), is associated not only with respiratory illness but also with profound vascular and coagulation disturbances. Long COVID (LC) is characterized by persistent symptoms such as fatigue, dyspnea, cognitive impairment, and palpitations. Mechanistically, SARS-CoV-2 induces direct endothelial injury, promotes a pro-inflammatory cytokine milieu, and activates platelets, leading to immunothrombosis and impaired fibrinolysis. Consequently, patients exhibit microthrombosis, elevated plasma D-dimer, fibrinogen dysregulation, and persistent hypercoagulability. Clinically, this translates into an increased risk of venous thromboembolism, including deep vein thrombosis and pulmonary embolism, as well as arterial thrombotic events such as myocardial infarction and stroke, which may persist months after acute infection. Understanding the interplay between endothelial injury, inflammation, and coagulation is crucial for risk stratification and the development of preventive and therapeutic strategies. We conducted a systematic narrative review of the literature, including human clinical and mechanistic studies identified through PubMed, Scopus and Web of Science up to 30 September 2025. This review synthesizes current evidence on vascular complications in LC, highlighting endothelial dysfunction as a central pathophysiological nexus linking the acute phase of SARS-CoV-2 infection with chronic LC manifestations.

## 1. Introduction

Coronavirus disease 2019 (COVID-19) is an infectious disorder caused by the severe acute respiratory syndrome coronavirus 2 (SARS-CoV-2), a single-stranded RNA virus belonging to the *Coronaviridae* family. The initial outbreak was identified in Wuhan, China, in December 2019, and the World Health Organization (WHO) declared a global pandemic in March 2020. Since then, more than 625 million confirmed cases and over seven million fatalities have been documented worldwide [1,2,3].

SARS-CoV-2 infection exhibits a broad clinical spectrum, ranging from asymptomatic or mild respiratory illness to severe and life-threatening multisystem involvement. The initial manifestations most frequently include dry cough, sore throat, rhinorrhea, dyspnea, fever, fatigue, and various gastrointestinal disturbances. Based on clinical severity, the disease is stratified into mild (without radiographic evidence of pneumonia), moderate (fever and respiratory symptoms with pneumonia) and severe forms characterized by marked dyspnea, respiratory rate > 30/min and oxygen saturation < 93%. In critically ill individuals, the disease frequently progresses to acute respiratory distress syndrome, multiorgan dysfunction syndrome, or septic shock [4,5,6].

Several demographic and comorbid conditions have been associated with a higher risk of adverse outcomes, including advanced age (>60 years), arterial hypertension, diabetes mellitus, chronic obstructive pulmonary disease, chronic kidney disease, malignancies, immunodeficiency and obesity [7,8]. Among the numerous systemic complications of COVID-19, coagulopathy has emerged as a hallmark of severe disease, substantially contributing to thrombotic events, multiorgan injury and elevated mortality [9,10]. COVID-19–associated coagulopathy represents a distinct, multifactorial thromboinflammatory state that integrates immune dysregulation, endothelial injury and hypercoagulability. The underlying mechanisms are that SARS-CoV-2 induces persistent endothelial dysfunction and disturbances in immunothrombotic balance, contributing to both microvascular and macrovascular thrombosis [11,12].

LC denotes a constellation of persistent or newly emerging symptoms and clinical conditions that develop following the acute phase of SARS-CoV-2 infection [5,6]. According to the WHO definition (2023), LC is characterized primarily by symptoms lasting beyond three months after acute infection, without an alternative clinical explanation [6]. According to the US 2024 National Academies of Sciences, Engineering, and Medicine (NASEM) definition, LC also encompasses conditions, not just symptoms [3]. Although definitions differ across studies, with symptom duration cut-offs ranging from 4 to 12 weeks after acute infection [6,10]. In this review, the term LC is used consistently to encompass both persistent symptoms and clinically relevant conditions, in line with the mechanistic and vascular focus of this manuscript.

Predominant manifestations include chronic fatigue, exertional intolerance, dyspnea, cognitive dysfunction (“brain fog”), myalgia, and palpitations, while thrombotic events represent clinical sequelae or conditions rather than symptoms [7,12]. These symptoms can also be multifactorial and caused by mechanisms other than endothelial activation. They reflect sustained endothelial activation, low-grade chronic inflammation, and microcirculatory dysfunction.

Epidemiological studies estimate that approximately 10–20% of individuals recovering from acute COVID-19 develop LC [6,7,10]. The prevalence of LC varies across viral variants and pandemic waves, with reported rates that can reach up to 45% among hospitalized patients and 10–15% in non-hospitalized cohorts, though percentages vary across studies [12,13]. According to recent estimates, approximately 400 million individuals are affected by LC globally [14].

In a substantial subset of patients, LC is increasingly recognized as a state of persistent endothelial activation, microthrombosis, and dysregulated haemostasis, representing a pathophysiological continuum that bridges acute COVID-19–associated coagulopathy and chronic vascular complications.

## 2. Methods

### 2.1. Review Design

We conducted a systematic narrative review to synthesize current evidence on vascular dysfunction, coagulopathy, and thromboembolic complications associated with post-acute sequelae of SARS-CoV-2 infection (PASC), hereafter referred to as LC. The objective was to provide a comprehensive, mechanistic, and clinically oriented synthesis of the literature. Given the significant heterogeneity in study designs, populations, and outcome measures, a formal meta-analysis was deemed unsuitable.

### 2.2. Eligibility Criteria

Study eligibility was defined using the PICOS framework:

Population: Adults (≥18 years) with Long COVID (LC), encompassing individuals with persistent or newly emerging symptoms or clinically relevant conditions following acute SARS-CoV-2 infection. For search completeness, studies referring to post-acute sequelae of SARS-CoV-2 infection (PASC) were included, provided that LC was the primary condition investigated. Eligible studies defined symptom persistence beyond 4 weeks after acute infection, while also incorporating those applying the WHO post-COVID-19 condition definition (≥12 weeks).

Exposure/Context: Post-acute phase of SARS-CoV-2 infection.

Comparator: Healthy controls, individuals recovered from COVID-19 without reported long-term sequelae, or patients with other chronic conditions (e.g., myalgic encephalomyelitis/chronic fatigue syndrome).

Outcomes:

○ Indicators of endothelial dysfunction (e.g., vascular reactivity, endothelial biomarkers);
○ Evidence of coagulopathy (e.g., D-dimer, fibrinogen, hypercoagulability assays);
○ Markers of immunothrombosis (e.g., neutrophil extracellular traps (NETs), platelet activation markers);
○ Clinical thromboembolic events (e.g., deep vein thrombosis (DVT), pulmonary embolism (PE)).

Study Types: Original observational studies (prospective/retrospective cohorts, case-control, cross-sectional), clinical trials, and mechanistic/laboratory studies in human participants.

Exclusion criteria: case reports, editorials, non-peer-reviewed preprints, and non-English publications.

Eligibility criteria for study inclusion were predefined using the PICOS framework and are summarized in Table 1.

### 2.3. Information Sources and Search Strategy

We systematically searched PubMed, Scopus, and Web of Science from inception to 30 September 2025. The search strategy employed a combination of Medical Subject Headings (MeSH) terms and keywords related to “COVID-19,” “Long COVID,” “vascular dysfunction,” “endothelial dysfunction,” “coagulopathy,” “antiphospholipid antibodies,” “cytokine storm,” and related concepts. To minimize the risk of missing relevant studies, multiple synonymous terms for LC were used, including ‘post-acute sequelae of SARS-CoV-2 infection’, ‘post-COVID condition’, and related descriptors. A full reproducible PubMed search strategy is provided in Supplementary Materials (File S1). Furthermore, reference lists of included studies and relevant systematic reviews were manually screened for additional eligible publications.

### 2.4. Study Selection Process

All search results were imported into EndNote (version 21, Clarivate Analytics) for deduplication. Two reviewers independently conducted a two-stage screening process:

Title and Abstract Screening: Studies were assessed against inclusion criteria based on their titles and abstracts.

Full-Text Review: The full texts of articles that passed the initial screening were retrieved and assessed in detail to determine final inclusion.

**Figure 1:**
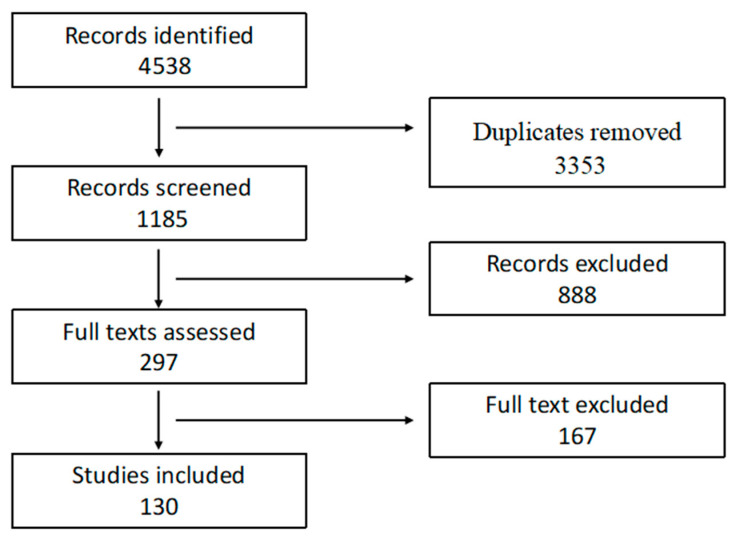
PRISMA diagram.

Any discrepancies between the reviewers were resolved through discussion or consultation with a third senior reviewer. The entire study selection process followed PRISMA (Preferred Reporting Items for Systematic Reviews and Meta-Analyses) guidelines and is summarized in Figure 1.

### 2.5. Data Extraction and Quality Assessment

Two reviewers independently extracted data from included studies using a standardized form. The extracted information included first author, publication year, study location, design, sample characteristics (including definition of LC), outcome measures, and key findings related to endothelial dysfunction, coagulation abnormalities, and thromboembolic complications.

### 2.6. Data Synthesis

Due to heterogeneity across study methodologies, a narrative synthesis approach was used. Findings were organized into four predefined thematic domains:

1. Endothelial injury and vascular dysfunction;
2. Coagulation and fibrinolytic dysregulation;
3. Immunothrombosis and platelet activation;
4. Clinical thromboembolic events: incidence and risk factors.

Within each domain, we evaluated the strength, consistency, and mechanistic plausibility of the evidence, integrating molecular, physiological, and clinical observations. Emphasis was placed on translational relevance to vascular risk stratification and preventive strategies in LC.

## 3. Pathophysiology of Vascular Complications in Long COVID

The pandemic of infection caused by SARS-CoV-2, the etiologic agent of COVID-19, has shown that the virus affects not only the respiratory system but also haemostasis, the endothelium and the microcirculation [12]. One of the most important systemic manifestations of the disease is COVID-19-associated coagulopathy, which substantially contributes to morbidity and mortality, particularly in severe forms of the disease.

COVID-19-associated coagulopathy is the result of a complex interaction between inflammation, endothelial dysfunction, platelet activation and activation of the coagulation pathways [15]. COVID-19-related coagulopathy displays features of immunothrombosis, as it arises from activation of the coagulation system as part of the immune response [15,16].

### 3.1. Long COVID and Endothelial Dysfunction

LC, as defined across different clinical and research frameworks, is a multi-organ syndrome whose aetiology is still not fully elucidated. The condition comprises a wide spectrum of symptoms that persist after recovery from the acute phase of COVID-19. More than 200 symptoms have been described across studies, affecting almost every organ system in the body [17,18]. This heterogeneity indicates that LC cannot be explained by dysfunction of a single organ or organ system, but most likely reflects a disturbance at the level of fundamental regulatory mechanisms of homeostasis. Endothelial dysfunction is increasingly recognized as an important pathophysiological mechanism that links the vascular, inflammatory and microcirculatory changes observed in patients with LC [19,20]. In this review we primarily focus on phenotypes of LC in which vascular, endothelial and haemostatic disturbances are prominent.

#### 3.1.1. Endothelial Susceptibility to SARS-CoV-2

Endothelial cells are known to be susceptible to SARS-CoV-2, and the roles of angiotensin-converting enzyme 2 (ACE2) and transmembrane serine protease 2 (TMPRSS2) are key to understanding the vascular complications of COVID-19. Endothelial cells express ACE2, which serves as the primary receptor for SARS-CoV-2 and thereby facilitates viral entry. TMPRSS2, on the other hand, proteolytically activates the S-protein, enabling fusion of the viral and cellular membranes and leading to infection [21]. Endothelial injury has been attributed in some studies to direct viral interaction or infection, whereas other evidence suggests predominantly indirect, immune-mediated mechanisms, resulting in endotheliitis, vascular inflammation and multiorgan involvement [22].

**Figure 2:**
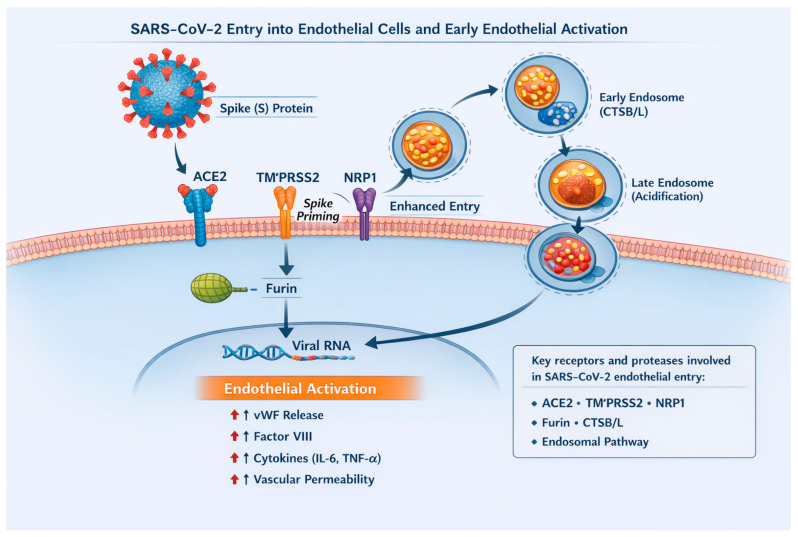
SARS-CoV-2 entry into endothelial cells and early endothelial activation.

In addition, interaction between SARS-CoV-2 and ACE2 may disturb the balance of the renin–angiotensin system and reduce ACE2 availability, which in turn increases angiotensin II activity, promoting vasoconstriction, inflammation and further cardiovascular damage in patients with COVID-19 [23]. Beyond its direct cytopathic effect, SARS-CoV-2 can induce a strong immune response that secondarily damages the endothelium. During the “cytokine storm”, molecules such as interleukin (IL)-6, tumor necrosis factor-α (TNF-α) and IL-1β can increase endothelial permeability and activate leukocytes [24]. The complement system, particularly the terminal C5b-9 complex, can further compromise the integrity of the endothelial layer [25]. NETs can create a highly thrombogenic microenvironment activate platelets and procoagulant factors, while disturbance of the renin–angiotensin system with predominance of angiotensin II promotes vasoconstriction, increases oxidative stress, and reduces nitric oxide (NO) bioavailability [26,27]. Together, these mechanisms may disrupt endothelial homeostasis and, in some individuals, may persist beyond apparent clinical recovery, defined as resolution of acute infection-related symptoms despite ongoing subclinical vascular dysfunction [22,28]. The key mechanisms involved in SARS-CoV-2 entry into endothelial cells and the resulting early endothelial activation are summarized in Figure 2.

Schematic representation of the mechanisms involved in SARS-CoV-2 entry into endothelial cells and the subsequent early endothelial activation. The viral spike (S) protein binds to angiotensin-converting enzyme 2 (ACE2) expressed on the endothelial surface, while transmembrane serine protease 2 (TMPRSS2) and furin facilitate spike protein priming and membrane fusion. Neuropilin-1 (NRP1) may further enhance viral entry. Alternatively, SARS-CoV-2 can enter endothelial cells via the endosomal pathway, where cathepsins B and L (CTSB/L) and endosomal acidification contribute to viral processing and release of viral RNA into the cytoplasm.

#### 3.1.2. Endothelial Dysfunction in Long COVID

Endothelial dysfunction is a frequently reported feature of LC. It is commonly characterized by reduced NO bioavailability and impairment of the eNOS–NO signaling pathway, potentially driven by persistent inflammation and oxidative stress [29,30]. Cytokines released during the inflammatory response to SARS-CoV-2, such as TNF-α and IL-1β, can reduce NOS expression and increase arginase activity, leading to an imbalance in arginine metabolism and decreased NO production [31]. Reduced NO availability may consequently contribute to increased vascular tone, impaired vasodilation and a prothrombotic milieu. The study by Munteanu et al. demonstrated that LC is clearly associated with endothelial dysfunction, with frequently increased vascular tone and impaired flow-mediated vasodilation (FMD) [32]. Reduced NO bioavailability, driven by IL-6-mediated inflammation and oxidative stress, disrupts endothelium-dependent vasodilation and increases vascular resistance [27]. Superoxide radicals (O_2_^−^) generated during inflammation may react with NO to form peroxynitrite (ONOO^−^), a highly reactive molecule capable of damaging endothelial proteins and lipids. Oxidative stress, characterized by excessive production of reactive oxygen species (ROS), contributes substantially to endothelial dysfunction and microvascular injury and may persist beyond the acute phase of SARS-CoV-2 infection [33,34].

#### 3.1.3. Microinflammation and Endothelial Activation

Elevated levels of von Willebrand factor (vWF) and factor VIII have been observed both in the acute and in patients with LC. Such increases in these molecules clearly contribute to endothelial activation and the development of prothrombotic states characteristic of COVID-19. Higher vWF and factor VIII levels have been associated with disease severity and are likely to contribute to the persistent vascular complications observed in LC [35,36]. Data on Intercellular Adhesion Molecule 1 (ICAM-1) and Vascular Cell Adhesion Molecule 1 (VCAM-1) in LC are still limited. At present, these markers should be regarded as indicators of endothelial activation in research settings rather than validated clinical biomarkers for LC. Persistent endothelial activation is evident during both the acute SARS-CoV-2 infection and the LC phase. As is known, up-regulation of these adhesion molecules promotes leukocyte adhesion and transmigration, driving vascular inflammation and contributing to microvascular injury [37]. It is also possible that P-selectin is released from activated platelets in COVID-19, thereby facilitating platelet–leukocyte and platelet–endothelium interactions and further amplifying thrombo-inflammatory processes [38]. In children with LC, increased platelet P-selectin expression has likewise been reported, indicating persistent platelet activation and endothelial activation [39]. Collectively, these findings indicate a persistently activated endothelial phenotype, which may contribute to symptoms such as fatigue, tachycardia, dyspnea and cognitive complaints.

#### 3.1.4. Glycocalyx Damage

The glycocalyx is a microscopic structure that lines the vascular endothelium. It is involved in the regulation of mechanotransduction and permeability and possesses anticoagulant properties. LC is characterized by persistent symptoms following SARS-CoV-2 infection and is associated with several pathophysiological mechanisms, including degradation of the endothelial glycocalyx. This degradation arises as part of the inflammatory response triggered by the SARS-CoV-2 spike protein; the spike protein may persist in the body and interact with various receptors, leading to inflammation and endothelial injury [40,41]. The glycocalyx, therefore, plays an important role in maintaining microvascular integrity. Symptoms such as fatigue and cognitive impairment in LC may be explained, at least in part, by glycocalyx degradation [42]. Glycocalyx breakdown in LC can be driven by activation of oxidative stress and increased release of inflammatory cytokines. Supporting this are data showing elevated levels of inflammatory markers such as IL-6, particularly in patients with LC [43]. However, current evidence is derived mainly from small and methodologically heterogeneous studies, and direct clinical assessment of glycocalyx integrity remains challenging.

#### 3.1.5. Microthrombosis and Impaired Microcirculation

Increased thrombogenicity and microthrombus formation have been reported not only in acute COVID-19 but also in LC. Persistent inflammation, endothelial dysfunction and platelet hyper-reactivity may contribute to a sustained prothrombotic state [29]. Some studies have reported an increased risk of thromboembolic events persisting for up to 49 weeks after infection, although longer persistence cannot be excluded due to limited follow-up data [34]. These abnormalities have been linked to increased thrombin generation and impaired fibrinolysis, partly mediated by elevated plasminogen activator inhibitor-1 (PAI-1). PAI-1 is a key endogenous inhibitor of tissue plasminogen activator (tPA) and urokinase plasminogen activator (uPA), and its persistent up-regulation results in suppression of fibrinolysis, favoring fibrin accumulation and microthrombus persistence. Beyond its antifibrinolytic effects, PAI-1 exerts direct pro-inflammatory and pro-thrombotic actions on the endothelium, promoting endothelial activation, cellular senescence, and increased expression of adhesion molecules [44,45]. Elevated PAI-1 levels have been associated with microvascular obstruction and impaired capillary perfusion, thereby contributing to sustained microcirculatory dysfunction observed in LC. Chronic low-grade inflammation, driven by cytokines such as IL-6 and transforming growth factor-β (TGF-β), may further stimulate endothelial PAI-1 expression, creating a self-perpetuating cycle of endothelial dysfunction, impaired fibrinolysis, and microvascular thrombosis [45]. Accordingly, prolonged clinical surveillance of selected high-risk patients may be warranted, and in carefully selected cases, extended thromboprophylaxis may be considered in line with emerging evidence and evolving guideline recommendations.

### 3.2. COVID-19 and Coagulation

#### 3.2.1. Immunothrombosis

Immunothrombosis in COVID-19 and LC is reflected by a distinct set of circulating biomarkers that capture the interplay between inflammation, endothelial activation, coagulation, and platelet activation [46]. Key biomarkers of immunothrombosis include elevated D-dimer and fibrinogen levels, endothelial activation markers such as vWF and factor VIII, components of NETs, and indices of platelet activation. While several of these parameters are discussed in detail in the coagulation system, their coexistence reflects a coordinated immunothrombotic response rather than isolated abnormalities of the coagulation cascade.

**Figure 3:**
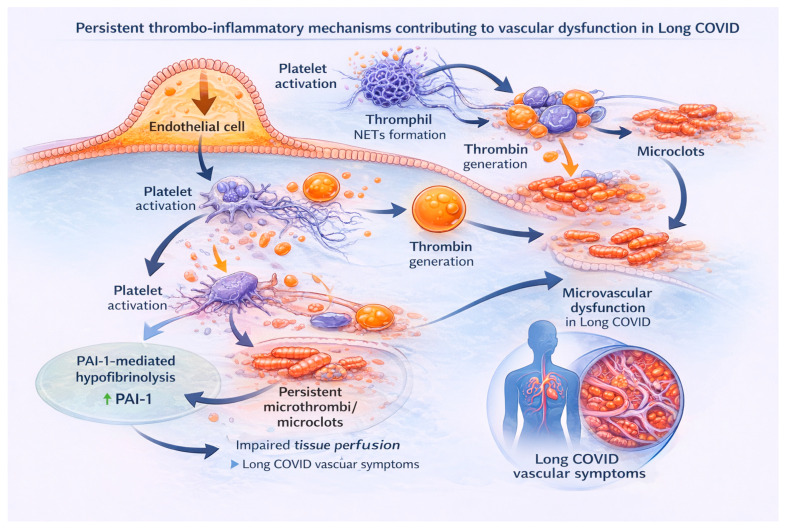
Persistent thrombo-inflammatory mechanisms contributing to vascular dysfunction in Long COVID.

In parallel with endothelial activation, a pronounced inflammatory response develops, accompanied by the release of pro-inflammatory cytokines that further damage the endothelium, induce tissue factor expression on monocytes and macrophages, and promote platelet activation [47]. Activated platelets release pro-inflammatory and procoagulant mediators, including serotonin, thromboxane A2 and CD40 ligand, and form aggregates with leukocytes, thereby further amplifying the inflammatory cascade. Neutrophils in this process generate NETs that entrap platelets and fibrin and contribute to microthrombus formation [46,48]. NETs can be quantified in plasma by measuring circulating myeloperoxidase–DNA (MPO–DNA) complexes, among other approaches, which serve as a widely used surrogate biomarker of NETs formation in inflammatory and thrombotic conditions [4,49]. In this way, inflammation and coagulation become tightly interconnected processes, collectively referred to as immunothrombosis (Figure 3). Importantly, immunothrombosis begins during the acute phase of SARS-CoV-2 infection and, according to several studies, may persist beyond viral clearance, contributing to the vascular complications seen in LC.

This figure illustrates the downstream thrombo-inflammatory pathways that may persist after the acute phase of SARS-CoV-2 infection and contribute to vascular dysfunction in LC. Endothelial activation promotes platelet activation and interaction with leukocytes, including neutrophils, leading to neutrophil extracellular trap (NETs) formation. Activated platelets and NETs enhance thrombin generation and fibrin formation, resulting in persistent microthrombi and microclots within the microcirculation. Elevated plasminogen activator inhibitor-1 (PAI-1) contributes to hypofibrinolysis, limiting fibrin degradation and favoring microclot persistence. These processes impair tissue perfusion and oxygen delivery, ultimately leading to chronic microvascular dysfunction and the vascular symptoms observed in LC.

#### 3.2.2. The Coagulation System in COVID-19

Activation of the coagulation system in COVID-19 occurs predominantly via the extrinsic pathway, as pro-inflammatory cytokines have been shown to induce tissue factor expression on monocytes and macrophages, leading to activation of factor VII and thrombin generation [50]. Thrombin not only converts fibrinogen to fibrin but also acts as a potent pro-inflammatory mediator that further stimulates cytokine production and platelet activation. At the same time, inflammatory cytokines stimulate hepatic synthesis of fibrinogen, resulting in elevated fibrinogen levels, particularly in the early stage of disease [51,52]. Initially, a hypofibrinolytic state has been reported, driven by increased PAI-1 activity and reduced plasmin generation. In some patients, progressive consumption of coagulation factors and worsening systemic inflammation may later result in declining fibrinogen levels, reflecting transition toward a consumptive phase of coagulopathy secondary to hyperfibrinolysis [53]. This dynamic imbalance contributes to haemostatic instability, with simultaneous tendencies toward thrombosis and bleeding.

Microthrombosis and fibrin deposition lead to increased fibrinolytic activity, resulting in the generation of fibrin degradation products (FDPs) and D-dimer [54,55]. Elevated D-dimer levels have consistently been reported as sensitive laboratory markers of COVID-19-associated coagulopathy and correlate with disease severity and mortality [56].

The most pronounced coagulation changes occur in the microcirculation, particularly in the lungs, where fibrin deposition and microthrombus formation impair oxygenation. Histopathological analyses have demonstrated diffuse microangiopathic thrombosis with prominent fibrin deposits in pulmonary capillaries [57]. Hypoxia may further exacerbate coagulation through activation of hypoxia-inducible factors (HIF-1α), which can stimulate tissue factor expression and suppress fibrinolysis via increased PAI-1 activity [58,59].

Elevated fibrinogen forms part of the acute-phase response to IL-6, whereas prolonged prothrombin time (PT) may be observed as partial consumption of coagulation factors [12]. PT may be normal or only mildly prolonged at the beginning of the disease, but as severe inflammation progresses, it becomes more prolonged because of factor consumption and impaired hepatic synthesis under the influence of cytokines. At the same time, activated partial thromboplastin time (aPTT) may remain normal, indicating that COVID-19-associated coagulopathy is not a typical disseminated intravascular coagulation syndrome, but rather a condition in which hypercoagulability predominates with localized factor consumption [12,60]. Unlike classic disseminated intravascular coagulation, which is often dominated by bleeding manifestations, COVID-19-associated coagulopathy is characterized by a predominantly prothrombotic phenotype with relatively preserved platelet counts and only modest prolongation of coagulation times.

In COVID-19 infection, platelets undergo activation and aggregation mediated by cytokines and endothelial injury. Activated platelets release procoagulant microparticles and increase synthesis of thromboxane A2 and serotonin, thereby further promoting vasoconstriction and aggregation. Although platelet counts are often normal or only mildly reduced, their functional hyperactivity leads to increased thrombus formation. In more severe forms of the disease, platelet reserves may become depleted and thrombocytopenia may develop; this is usually moderate, but if it worsens rapidly, it indicates severe consumption and an unfavorable prognosis [61].

Antithrombin, the main physiological inhibitor of thrombin and factor Xa, has been reported to be reduced in COVID-19. Its decline is due to a combination of consumption in the setting of intense coagulation, reduced hepatic synthesis and losses through damaged capillaries in severe inflammation. Reduced antithrombin levels further contribute to maintaining a procoagulant state and to decreased efficacy of heparin therapy [62].

The roles of factor VIII and vWF in the pathogenesis of COVID-19-associated coagulopathy are particularly important. Under the influence of inflammatory cytokines, endothelial cells release large amounts of vWF and factor VIII, thus increasing their plasma concentrations and creating a tendency towards thrombosis. High levels of vWF and factor VIII are characteristic of endothelial activation and injury and provide further evidence that the endothelium is a central target in this disease. Increased vWF simultaneously promotes platelet adhesion and the formation of platelet–fibrin plugs in the microcirculation, which is corroborated histologically by findings of microangiopathic thrombosis in the lungs, kidneys and brain [62,63]. Alterations in vWF and ADAMTS13 have also been described. The ADAMTS13 protease regulates vWF activity by cleaving vWF multimers; therefore, an imbalance between vWF and ADAMTS13 may contribute to the development of thrombotic disorders [64]. An increase in vWF levels on the one hand and a decrease in ADAMTS13 on the other thus have additional thrombogenic significance [65].

In patients who have recovered from SARS-CoV-2 infection, several studies have reported the presence of antiphospholipid antibodies (aPL), including anticardiolipin (aCL), anti-β2-glycoprotein I (anti-β2GPI) and lupus anticoagulant (LA) antibodies [66,67]. Their presence has been linked to an increased risk of arterial and venous thrombosis, although the underlying mechanism may be transient and immunologically non-specific [67]. Viral infection is thought to provoke polyclonal B-lymphocyte activation and production of autoantibodies, including aPL, as part of the immune response to acute inflammation [68]. These antibodies can bind phospholipid-dependent proteins on the surface of endothelial cells and platelets, leading to their activation and the creation of a procoagulant milieu [69].

Collectively, increased coagulation activation, impaired anticoagulant pathways, and hypofibrinolysis create a state known as thromboinflammation [70]. The SARS-CoV-2 virus, through endothelial injury and the inflammatory cascade, initiates a vicious cycle in which thrombin, cytokines, and activated platelets mutually stimulate one another (Table 2). Thrombin and FDP further activate inflammatory cells, while inflammatory cytokines enhance coagulation [12,50,51]. The result is a dynamic imbalance in which the processes of thrombosis and fibrinolysis occur simultaneously, explaining the concurrent presence of elevated D-dimer, fibrinogen, FDP, and a moderate decrease in antithrombin and platelets [71].

Taken together, these changes explain the characteristic laboratory profile of COVID-19-associated coagulopathy: elevated D-dimer, moderately prolonged PT, initially raised fibrinogen that may later fall, the presence of FDP, mild thrombocytopenia and reduced antithrombin, together with increased factor VIII and vWF levels (Table 3). These parameters reflect combined activation, consumption and inflammatory stimulation of the coagulation system, thereby explaining how SARS-CoV-2 leads to coagulopathy that significantly contributes to disease severity and mortality. Clinically, this manifests as an increased risk of venous thromboembolism, microthrombosis in the lungs, kidneys and brain, and arterial thromboses [72].

Importantly, immunothrombosis and endothelial activation initiated during the acute phase of SARS-CoV-2 infection may persist beyond viral clearance in a subset of patients and are increasingly recognized as key mechanisms underlying the persistent vascular and coagulation abnormalities observed in LC [11,73].

### 3.3. Persistent Vascular Disturbances and Coagulopathy in Long COVID

Following the acute phase of SARS-CoV-2 infection, a subset of patients develops LC, characterized by persistent or newly emerging symptoms and conditions. Importantly, immunothrombosis and thrombo-inflammatory pathways initiated during the acute phase of SARS-CoV-2 infection (Figure 3) may persist beyond viral clearance and contribute to the vascular complications seen in LC. Clinically, LC may manifest as prolonged fatigue, dyspnea, cognitive difficulties, myalgia and other symptoms. Increasing evidence suggests that persistent endothelial dysfunction and haemostatic dysregulation may underlie these manifestations [74,75]. Multiple observational studies have reported signs of ongoing coagulation activation in LC patients, even in the absence of clinically overt thrombosis [76,77].

Endothelial cells that have undergone viral injury and cytokine stimulation may retain a pro-inflammatory and procoagulant phenotype even after the virus has been cleared [22]. Elevated circulating levels of endothelin-1, ICAM-1, VCAM-1 and soluble thrombomodulin have been documented months after infection, indicating sustained endothelial activation [18,55,78].

Prolonged endothelial dysfunction may lead to microcirculatory impairment in organs such as the lungs, heart and brain, contributing to symptoms including dyspnea, visual disturbances, and reduced exercise capacity [79]. Microthrombotic changes and subclinical microangiopathy have been described in selected studies, supporting the concept of prolonged thromboinflammatory activity [80].

In LC, subclinical activation of immunothrombosis may occur, with ongoing low-grade activation of platelets and leukocytes. Persistent NETs and circulating microparticles rich in tissue factor have been described, maintaining a low but chronic activation of coagulation pathways [74,81]. Such a state may contribute to prolonged vascular damage, microembolisation and endothelial dysfunction even in the absence of overt thrombosis.

LC has been associated in several studies with a hypofibrinolytic state, in part mediated by elevated PAI-1 levels. Increased PAI-1 inhibits plasmin generation, limits fibrin degradation, and favors fibrin persistence within the microcirculation [73]. Small fibrin aggregates enriched with α2-antiplasmin and complement components (“microclots”) have been identified in the plasma of some patients months after infection and appear resistant to fibrinolysis [82,83]. Although these abnormalities have been documented for many months, their potential persistence beyond currently available follow-up periods cannot be excluded.

aPL has been detected in a proportion of patients during COVID-19 and in LC [75,77]. Such autoimmune activation helps sustain an inflammatory and prothrombotic state, prolonging the duration of vascular complications. In the acute phase of COVID-19, the prevalence of positive aPL is between 30% and 50%, but later studies have shown that in most cases the titres are low and transient [84,85]. However, in a subset of patients, particularly those with more severe clinical presentations of LC, these antibodies may persist for many months and may be associated with long-term endothelial damage and microthrombosis [76]. Recent meta-analyses suggest that the presence of aPL may be a marker of prolonged inflammatory activity, but is not always a predictor of thrombosis in the absence of additional risk factors. It is therefore recommended that their presence be interpreted in the context of the clinical picture and other haemostatic parameters [55,75]. Routine screening for aPL in all individuals with LC is therefore not currently justified, but targeted testing may be appropriate in patients with unexplained thrombotic events or other autoimmune features.

Taken together, these findings indicate that LC reflects prolonged dysregulation of endothelial–coagulation homeostasis, with sustained interaction between inflammation, immune activation, and thrombosis even after resolution of acute infection. Understanding these mechanisms is essential for improved vascular risk stratification and the development of targeted preventive and therapeutic strategies.

## 4. Complications of Long COVID

### 4.1. Venous Thromboembolic Complications

Besides the respiratory manifestations that may lead to long-term disability, hemostatic abnormalities represent a major complication in patients with severe or critical COVID-19. These alterations reflect a prothrombotic state driven by endothelial dysfunction, inflammation, and immune-mediated mechanisms. Thrombotic events associated with COVID-19 are predominantly venous thromboembolic (VTE) events, including DVT and PE [86,87]. Critically ill patients are frequently immobilized in the Intensive Care Unit due to acute respiratory failure, which further increases the risk of VTE. This risk is reflected by commonly used clinical scores such as the Padua Prediction Score (bed rest: 3 points, infection: 1 point, respiratory failure: 1 point) [88]. In the context of LC, persistent endothelial dysfunction and immune-mediated coagulopathy may maintain a prothrombotic milieu even after acute resolution of the acute infection [88,89,90].

In addition to pulmonary embolism, DVT can present as a frequent manifestation of VTE in LC. However, some studies suggest a weaker association between DVT and PE in LC cohorts, potentially because pulmonary microthrombi may arise from in situ thrombosis rather than classic embolization from peripheral veins [91]. Histopathological analyses indicate that pulmonary emboli and pulmonary microthrombi differ in composition: emboli typically resemble distal venous thrombi, whereas pulmonary microthrombi contain a higher proportion of platelets and fibrin. These differences may have important implications for therapeutic and prophylactic strategies [86]. Furthermore, studies have reported persistent plasma microthrombi resistant to fibrinolysis in patients with LC, which may contribute to prolonged symptoms [91]. Established comorbidities, including cardiovascular disease, diabetes, obesity, and hypertension, should be considered when assessing thromboembolic risk, with a higher prevalence reported in immunocompromised populations [91].

During the acute phase of COVID-19, the prevalence of VTE has been reported to approach 30%, with DVT occurring in approximately 20% and PE in 18% of hospitalized patients [86,92]. Increasing age is a recognized risk factor for thromboembolic complications. In a retrospective cohort study with follow-up of up to 30 days after hospital discharge, thrombotic events-including PE, intracardiac thrombus, and ischemic stroke were observed in 2.5% of patients [93]. In tLC, survivors beyond 30 days after COVID-19 diagnosis demonstrated an increased risk of DVT (HR 1.98; 95% CI 1.94–2.24), PE (HR 2.93; 95% CI 2.73–3.15), and superficial vein thrombosis (HR 1.95; 95% CI 1.80–2.12) [94]. These findings suggest that LC may be associated with an increased risk of VTE and underscore the importance of early recognition and risk stratification.

Patients with underlying chronic hypercoagulable states appear more likely to develop severe COVID-19 and subsequently LC. The coexistence of pre-existing hypercoagulability and the acquired prothrombotic state associated with LC may further increase thromboembolic risk [95]. Moreover, patients demonstrating hypercoagulable profiles on rotational thromboelastometry during active infection were reported to be 17–28 times more likely to develop LC in selected cohorts [96].

Among patients with LC, elevated D-dimer levels and increased circulating endothelial cells have been observed in approximately 25–30% of individuals up to four months post-infection [97]. Additional studies have reported increased levels of coagulation-related and proinflammatory markers, including factor Xa activity, platelet factor 4, vWF, homocysteine, aPL, and NETs, all of which may promote thrombus formation [95]. These observations support a close interaction between immune-inflammatory pathways and coagulation in LC. While this provides a rationale for investigating anticoagulant strategies in selected high-risk patients, robust randomized clinical trial data are currently lacking. At the same time, untreated coagulopathy during the acute phase may contribute to persistent tissue hypoxia and multiorgan manifestations characteristic of LC, highlighting the need for careful individual risk assessment [98,99].

The estimated incidence of VTE in LC ranges from 0.5–5.4%, which is approximately fourfold higher than that observed after other infectious conditions [99,100]. Identified risk factors include prolonged immobility, persistent hypoxia, advanced age, and obesity. Compared with non-infected individuals, LC patients demonstrated a higher risk of incident PE (HR 3.16, 95% CI 2.63–3.79, *p* < 0.0001) over a median follow-up of 8.5 months [21]. VTE and PE appear to be more common in the early period following SARS-CoV-2 infection, and some studies suggest a higher relative risk in women during LC, although the absolute risk in the general population remains relatively low [99].

Due to the limited and heterogeneous nature of available data, these findings cannot yet be directly translated into uniform recommendations for extended thromboprophylaxis in all LC patients. Nevertheless, increased vigilance and individualized assessment are warranted, particularly in high-risk populations such as elderly patients and those with persistent prothrombotic markers.

### 4.2. Arterial Thromboembolic Complications

Arterial complications represent a clinically significant component of LC and contribute substantially to long-term morbidity. Although the acute phase of SARS-CoV-2 infection is recognized for its high incidence of thrombosis, myocardial injury and arrhythmias, multiple cohort studies indicate that the risk of these events remains elevated well beyond the acute phase, persisting for months and in some cases longer than one year after infection [101]. The underlying pathophysiological mechanisms include chronic endothelial dysfunction, persistent inflammation, a procoagulant state, autonomic dysregulation and direct cardiomyocyte injury, which together induce structural and functional changes in the arterial wall, culminating in clinically manifest disorders such as increased arterial stiffness, de novo hypertension, peripheral arterial dysfunction and, less commonly, acute arterial thrombotic events. Experimental studies have shown that the SARS-CoV-2 spike protein can induce prolonged inflammatory activation of human endothelial cells, with increased expression of adhesion molecules, release of cytokines and chemokines, and a pro-coagulant phenotype [102]. Notably, these changes have also been observed in individuals without pre-existing cardiovascular disease, suggesting a SARS-CoV-2-specific vascular pathophysiological mechanism [103]. Identifying these complications, understanding their mechanisms and developing appropriate diagnostic and therapeutic strategies are therefore crucial to preventing long-term consequences.

#### 4.2.1. Major Adverse Cardiovascular Events and Acute Myocardial Infarction

Major adverse cardiovascular events (MACE)—including myocardial infarction, stroke and cardiovascular death—are significantly increased in LC. Evidence indicates that the risk extends beyond the period of acute illness and remains elevated during recovery, rather than being confined to the acute phase alone. Large cohort studies show that individuals who survive SARS-CoV-2 infection have an elevated risk of these events in the post-acute period (>30 days), and that this risk persists for at least one year following infection, even among those who were not hospitalized during the acute phase [94]. Long-lasting endothelial dysfunction, increased arterial stiffness and a persistent procoagulant state are considered to be key mechanisms contributing to this increased risk [103,104]. SARS-CoV-2 can directly damage cardiomyocytes (via ACE2 receptors), potentially resulting in micro-scarring that may predispose to atherosclerosis progression and arterial thrombotic events [105].

The main high-risk groups include patients with severe acute illness and/or pre-existing cardiovascular disease; however, studies also demonstrate a relative increase in absolute risk among younger and previously healthy individuals, compared with non-infected control populations. This observation underscores the need for vigilance and symptom monitoring—including chest pain, dyspnea, palpitations and new exercise intolerance—even in patients without traditional cardiovascular risk factors [106]. There is also evidence that arterial stiffness may remain elevated long after the acute infection, and arterial stiffness is a recognized predictor of cardiovascular events and mortality. In one study assessing vascular function over six months in young adults afte COVID-19, central arterial stiffness (carotid–femoral pulse wave velocity, cfPWV) decreased significantly during recovery, whereas carotid stiffness and related parameters remained persistently elevated compared with reference values. These findings suggest that central vascular function may normalize more rapidly, while peripheral vascular segments may sustain longer-lasting injury [107]. In some patients, additional factors such as persistent tachycardia due to autonomic dysfunction, worsening arterial stiffness and newly developed or aggravated cardiometabolic risk factors (e.g., hypertension and dyslipidaemia) may exacerbate myocardial oxygen supply–demand mismatch and precipitate acute myocardial infarction in LC [108]. Together with chronic inflammation, these mechanisms likely underlie the increased incidence of major cardiovascular events following SARS-CoV-2 infection.

#### 4.2.2. Ischaemic Stroke

Ischaemic stroke is a recognized, though relatively uncommon, late neurovascular complication of LC. Several large cohort studies demonstrate that individuals with prior SARS-CoV-2 infection have an increased risk of ischaemic stroke for up to one year or longer following infection [109]. The pathophysiology of ischaemic stroke in LC is multifactorial. SARS-CoV-2 can directly injure endothelial cells via ACE2 receptors, leading to endothelial inflammation and loss of antithrombotic properties. In parallel, systemic inflammation activates coagulation pathways and inhibits fibrinolysis, resulting in a sustained procoagulant state [110]. Long-term endothelial dysfunction in patients who have recovered from COVID-19 has been associated with elevated levels of vWF, factor VIII and other procoagulant molecules, while reduced protein C activity and increased inhibitory factors further disrupt haemostatic balance [111]. In addition, an increased incidence of atrial fibrillation has been reported in some LC cohorts, further elevating the risk of cardioembolic stroke [112].

#### 4.2.3. Cardiac Arrhythmias

Cardiac arrhythmias in LC represent a clinically and heterogeneous complication that may emerge months after the initial infection. The underlying mechanisms involve persistent inflammation, endothelial dysfunction and autonomic dysregulation, all of which may disturb normal cardiac electrophysiology [113]. Direct myocardial injury (for example, viral invasion and myocarditis via ACE2), together with fibrosis and electrolyte imbalance (e.g., hypokalaemia), can increase susceptibility to both atrial and ventricular arrhythmias [114]. A broad spectrum of arrhythmias—from atrial fibrillation to ventricular tachycardia—has been reported in LC. Some longitudinal studies suggest partial improvement or apparent resolution of arrhythmias over a two-year follow-up period; however, longer-term data remain limited, and incomplete recovery in certain patient subgroups cannot be excluded [115]. These observations highlight the importance of long-term rhythm surveillance. Importantly, many patients reporting palpitations exhibit benign rhythm disturbances, underscoring the need for careful clinical evaluation to differentiate low-risk findings from sustained or potentially life-threatening arrhythmias.

#### 4.2.4. Myocarditis

In LC, myocarditis represents an important inflammatory cardiac complication that may occur weeks to months after the initial infection. SARS-CoV-2 may directly invade cardiomyocytes and trigger persistent immune activation, leading to fibrotic myocardial remodeling [116]. Myocardial inflammation is often accompanied by elevated inflammatory markers, such as interleukins, and occasionally by increased troponin levels. Diagnosis is frequently established using non-invasive imaging modalities, particularly cardiac magnetic resonance imaging (MRI), which may reveal abnormalities in T1/T2 mapping and areas of myocardial fibrosis identified by late gadolinium enhancement (LGE) [117]. Other studies have shown that even in individuals who never experienced severe COVID symptoms, cardiac MRI frequently demonstrates a pattern of myocardial scarring (LGE) and alterations in ventricular function, while standard biomarkers (such as troponin) and electrocardiographic findings may still be normal [118]. These findings suggest that SARS-CoV-2 infection can induce subclinical but persistent myocardial injury, the long-term prognostic significance of which remains under investigation.

#### 4.2.5. Heart Failure

Heart failure is a well-documented cardiovascular sequela of LC and may develop as a downstream consequence of myocardial injury, including myocarditis and microvascular dysfunction. In a large cohort study (N3C; 587,330 hospitalized patients), COVID-19 hospitalization was associated with a 45% increased risk of developing de novo heart failure within one year of infection (HR 1.45) [119]. In some patients, myocarditis-related scarring and fibrosis may progress insidiously and remain clinically unrecognized until symptomatic heart failure becomes apparent [120]. Moreover, echocardiographic and imaging studies indicate that individuals with LC—including those without prior cardiovascular disease or conventional risk factors—may exhibit subtle impairments in left and right ventricular systolic and diastolic function. Although these parameters often remain within reference ranges, they differ significantly from those observed in non-infected control populations [121]. Together, these findings suggest that myocardial injury in LC may contribute to a spectrum of cardiac dysfunction, ranging from subclinical abnormalities to overt heart failure.

### 4.3. Microvascular and Peripheral Manifestations

Some individuals following the acute phase of COVID-19 report symptoms that persist for several weeks to months after the initial infection [87,122]. Pathophysiological mechanisms triggered by SARS-CoV-2 can also induce persistent microvascular alterations. Several studies suggest that SARS-CoV-2 activates coagulation pathways through procoagulant factors and proinflammatory cytokines, thereby increasing the risk of atherosclerotic plaque instability, thrombosis, and tissue ischemia [123]. These mechanisms appear to be shared by both acute COVID-19 and LC, and it has been demonstrated that circulating microclots may exhibit amyloid-like properties [73]. The role of ACE2 in the development of these complications remains a subject of ongoing investigation [124]. Furthermore, persistent elevation of biomarkers reflecting thrombotic activity within the microcirculation has been reported for several months following the acute phase of infection, supporting the concept of impaired fibrinolysis and sustained microthrombus formation in LC [73,125,126].

Viral persistence in tissue reservoirs has been proposed as one of the mechanisms underlying LC, leading to sustained proinflammatory responses that promote microvascular dysfunction, immune dysregulation, and chronic tissue hypoxia [127]. Nailfold video capillaroscopy (NVC) studies have demonstrated a reduced capillary density per linear millimeter in patients with LC [128]. Similarly, in patients with LC, non-specific microvascular abnormalities—including capillary dilatation and microhemorrhages—have been observed up to 18 months after infection [127]. In addition, some studies indicate that in antinuclear antibody (ANA)-positive individuals, SARS-CoV-2 may act as a trigger for immune-mediated endothelial damage, thereby contributing to persistent microvascular injury [73]. The presence of ANA suggests a pre-existing or infection-induced autoimmune milieu, which may amplify endothelial injury and contribute to the chronicity of microvascular dysfunction. Both microvascular and endothelial injury are thought to play a central role in the heterogeneous clinical manifestations of LC. Moreover, reduced levels and impaired functional capacity of endothelial progenitor cells may further compromise vascular repair mechanisms in these patients. Damage to the blood–brain barrier has been identified in patients presenting with neurocognitive symptoms, while loss of capillary density and reduced blood flow have also been demonstrated in the sublingual and retinal microcirculation, as well as impaired flow-mediated skin fluorescence [11,129,130].

Among the acral manifestations associated with LC, chilblain-like or pernio-like lesions—commonly referred to as “COVID toes”—are among the most frequently reported and typically occur in young, previously healthy individuals with mild acute disease. These lesions are characterized by erythematous papules and macules, with or without blistering, affecting acral regions. Clinically, chilblain-like lesions resemble digital vasculopathy observed in connective tissue diseases [131]. In most cases, the lesions resolve spontaneously within approximately two weeks without specific treatment. If lesions persist beyond 30 days, therapeutic options may include aspirin, topical corticosteroids, vasodilators, and prostacyclin analogs [132]. Several authors have proposed that chilblain-like lesions may represent a visible manifestation of a systemic microvascular process rather than an isolated cutaneous finding [133].

Disseminated microangiopathy with microthrombus formation has been proposed as a pathogenic mechanism underlying livedo reticularis observed as a late manifestation of COVID-19. These lesions are characterized by regular, non-fixed, violaceous patches forming a reticular pattern with pale central areas. Immunofluorescence studies have demonstrated deposition of complement component C4c within affected vessels, supporting immune-mediated vascular injury [134,135]. More recently, activation of the complement cascade—particularly deposition of the terminal C5b–9 complex—has been implicated as an additional contributor to persistent endothelial damage and microvascular dysfunction in LC [136].

Episodes of discoloration, numbness, or pain in the fingers and toes due to transient reductions in blood flow may represent Raynaud-like phenomena occurring in patients with LC. In response to cold exposure or emotional stress, affected acral areas may turn white or blue. Autonomic nervous system dysfunction has been proposed as a key mechanism contributing to these symptoms. There have also been isolated reports of Raynaud-like phenomena developing after COVID-19 vaccination, although such cases appear to be rare and a causal relationship has not been firmly established [137].

## 5. Conclusions

LC has emerged as a complex multisystem condition in which persistent vascular dysfunction appears to represent a key pathogenic component. Chronic inflammation, immune dysregulation, endothelial injury, and sustained activation of coagulation pathways collectively contribute to the development of microvascular and macrovascular abnormalities long after the resolution of acute infection. Mechanisms such as endotheliitis, activation of the complement cascade, formation of fibrinolysis-resistant microclots, platelet hyperactivation, and involvement of the autonomic nervous system promote impaired perfusion, tissue hypoxia, and a broad spectrum of clinical manifestations. Microvascular rarefaction, capillary dilatation, microhemorrhages, and reduced flow-mediated responses have been widely documented and correlate with neurological, cardiovascular, and acral symptoms observed in patients with LC.

Growing evidence suggests that the vascular component provides a unifying framework linking many of the persistent symptoms and complications associated with LC. A deeper understanding of these interconnected mechanisms is essential for early recognition, risk stratification, and individualized management of affected patients. Further research is needed to identify reliable biomarkers, determine individuals at greatest risk, and develop effective therapeutic strategies aimed at supporting endothelial recovery, modulating immune activation, and restoring microcirculatory function. Future interventional studies should clarify whether therapies targeting endothelial repair, microthrombotic burden, or autonomic imbalance can modify the long-term trajectory of LC, ideally within multidisciplinary care pathways involving cardiology, hematology, neurology, and rehabilitation services.

## Figures and Tables

**Table 1:** Eligibility criteria of included studies according to the PICOS framework.

PICOS Component	Eligibility Criteria
**Population (P)**	Adults (≥18 years) with Long COVID (LC), encompassing individuals with persistent or newly emerging symptoms or clinically relevant conditions following acute SARS-CoV-2 infection. For search completeness, studies referring to post-acute sequelae of SARS-CoV-2 infection (PASC) were included, provided that LC was the primary condition investigated. Eligible studies defined symptom persistence beyond 4 weeks after acute infection, while also incorporating those applying the WHO post-COVID-19 condition definition (≥12 weeks).
**Intervention/Exposure/Context (I)**	Post-acute phase of SARS-CoV-2 infection, irrespective of initial disease severity or hospitalization status.
**Comparator (C)**	Healthy control individuals; individuals recovered from COVID-19 without reported long-term sequelae; or patients with other chronic conditions used as comparator groups (e.g., myalgic encephalomyelitis/chronic fatigue syndrome). Studies without a comparator group were also considered when mechanistic outcomes were reported.
**Outcomes (O)**	One or more of the following outcomes: (1) markers of endothelial dysfunction (e.g., vascular reactivity, endothelial biomarkers); (2) evidence of coagulation or fibrinolytic abnormalities (e.g., D-dimer, fibrinogen, hypercoagulability assays); (3) markers of immunothrombosis (e.g., neutrophil extracellular traps, platelet activation markers, antiphospholipid antibodies); and/or (4) clinical thromboembolic events, including deep vein thrombosis and pulmonary embolism.
**Study design (S)**	Original human studies, including observational studies (prospective or retrospective cohort studies, case–control studies, cross-sectional studies), clinical trials, and mechanistic or laboratory-based studies conducted in human participants.
**Exclusion criteria**	Case reports, editorials, narrative opinions, non-peer-reviewed preprints, animal-only studies, and non-English language publications.

**Table 2:** Mechanisms of COVID-19-associated coagulopathy.

Mechanism	Key Processes	Main Mediators	Consequences
Endothelial dysfunction	Direct endothelial infection via ACE2 receptors; loss of antithrombotic function	von Willebrand factor, factor VIII, endothelin-1	Procoagulant state, microthrombosis
Inflammatory activation	Cytokine storm and expression of tissue factor on monocytes	IL-6, IL-1β, TNF-α, tissue factor	Thrombin activation, increased coagulation
Platelet activation	Platelet activation and aggregation due to cytokines and endothelial damage	Thromboxane A_2_, serotonin, CD40L	Microthrombosis, platelet consumption
Immunothrombosis (NETs formation)	Neutrophil activation and formation of NETs	Neutrophil extracellular traps, fibrin	Microangiopathic thrombosis
Imbalance of coagulation and fibrinolysis	Increased PAI-1, reduced plasmin activity	PAI-1, plasmin	Hypofibrinolytic state, fibrin accumulation
Autoimmune mechanisms	Presence of antiphospholipid antibodies	aCL, anti-β2GPI, LA	Arterial and venous thrombosis

ACE2—angiotensin-converting enzyme 2, IL—interleukin, TNF-α—tumor necrosis factor-α, NETs—neutrophil extracellular traps, PAI-1—plasminogen activator inhibitor 1, aCL—anticardiolipin, anti-β2GPI—anti-β2-glycoprotein I, LA—lupus anticoagulant.

**Table 3:** Typical laboratory changes in COVID-19-associated coagulopathy.

Parameter	Change	Clinical Significance
D-dimer	↑ elevated	Marker of fibrinolysis and microthrombosis; correlates with disease severity
Fibrinogen	↑ early phase, ↓ late phase	Reflects inflammation and consumption
Prothrombin time	Normal → slightly prolonged	Partial consumption of coagulation factors
Activated partial thromboplastin time	Normal or slightly shortened	Hypercoagulable state
Antithrombin	↓ decreased	Consumption and decreased synthesis; reduced heparin efficacy
Platelets	Normal → mildly decreased	Consumption and activation
vWF and factor VIII	↑ elevated	Endothelial activation and microangiopathy
ADAMTS13	↓ decreased	Imbalance with vWF, prothrombotic effect

vWF—von Willebrand factor; ↑ indicates an increase, ↓ indicates a decrease, and → indicates no significant change.

## Data Availability

The data used to support the findings of this study are available from the corresponding author upon request.

## Supplementary Materials

The following supporting information can be downloaded at https://www.mdpi.com/article/10.3390/ijms27010433/s1.

## Abbreviations

The following abbreviations are used in this manuscript:

COVID-19	Coronavirus Disease 2019
SARS-CoV-2	Severe Acute Respiratory Syndrome Coronavirus 2
Long COVID	LC
WHO	World Health Organization
NASEM	National Academies of Sciences, Engineering, and Medicine
PASC	Post-Acute Sequelae of SARS-CoV-2
NETs	Neutrophil Extracellular Traps
DVT	Deep Vein Thrombosis
PE	Pulmonary Embolism
MeSH	Medical Subject Headings
PRISMA	Preferred Reporting Items for Systematic Reviews and Meta-Analyses
ACE2	Angiotensin-Converting Enzyme 2
TMPRSS2	Transmembrane Serine Protease 2
IL	Interleukin
TNF-α	Tumor Necrosis Factor-α
NO	Nitric Oxide
FMD	Flow-Mediated Vasodilatation
O_2_^−^	Superoxide Radicals
ONOO^−^	Peroxynitrite
ROS	Reactive Oxygen Species
vWF	von Willebrand Factor
ICAM-1	Intercellular Adhesion Molecule 1
VCAM-1	Vascular Cell Adhesion Molecule 1
PAI-1	Plasminogen Activator Inhibitor 1
tPA	tissue Plasminogen Activator
uPA	urokinase Plasminogen Activator
TGF-**β**	Transforming Growth Factor-β
MPO-DNA	Myeloperoxidase-DNA
FDP	Fibrin Degradation Products
HIF-1α	Hypoxia-Inducible Factor
PT	Prothrombin Time
aPTT	Activated Partial Thromboplastin Time
aPL	Antiphospholipid Antibodies
aCL	Anticardiolipin
anti-β2GPI	anti-β2-Glycoprotein I
LA	Lupus Anticoagulant
VTE	Venous Thromboembolism
MACE	Major Adverse Cardiovascular Events
cfPWV	Carotid–Femoral Pulse Wave Velocity
MRI	Magnetic Resonance Imaging
LGE	Late Gadolinium Enhancement
NVC	Nailfold Videoocapillaroscopy
ANA	Antinuclear Antibodies
