# Supplementary material for: Vascular Complications of Long COVID—From Endothelial Dysfunction to Systemic Thrombosis: A Systematic Review
Source: Int J Mol Sci. 2025 Dec 31;27(1):433. doi: 10.3390/ijms27010433 (PMC12786942; doi:10.3390/ijms27010433)
Supplement: Supplementary file 1 [file ijms-27-00433-s001.zip › ijms-4043863-supplementary.pdf]

Supplementary Material – File S1: Detailed Search Strategy

for

“Vascular complications of Long COVID syndrome – from endothelial dysfunction to systemic thrombosis”

This document provides the full electronic search strategy as executed in PubMed on September 30th, 2025. The strategy was adapted for syntax and controlled vocabularies for Scopus and Web of Science.

1 | "post-acute COVID-19 syndrome"[Mesh] OR "COVID-19"[Mesh] OR "Long COVID"[tw] OR "post COVID"[tw] OR "post-COVID"[tw] OR "post-COVID syndrome"[tw] OR "post-COVID condition"[tw] OR "post-COVID sequelae"[tw] OR "post-acute COVID"[tw] OR "post-acute COVID syndrome"[tw] OR "PASC"[tw] OR "long haul COVID"[tw] OR "long-haul COVID"[tw]

2 | "Endothelium, Vascular"[Mesh] OR "Endothelial Cells"[Mesh] OR "Vascular Diseases"[Mesh] OR "endothelial dysfunction"[tw] OR "vascular dysfunction"[tw] OR "endothelial injury"[tw] OR "vascular injury"[tw] OR "endotheliopathy"[tw] OR "endothelial damage"[tw] OR "microvascular dysfunction"[tw] OR "vasculopathy"[tw]

3 | "Blood Coagulation Disorders"[Mesh] OR "Thrombosis"[Mesh] OR "Hypercoagulability"[tw] OR "coagulopathy"[tw] OR "prothrombotic"[tw] OR "hypercoagulable"[tw] OR "thromboinflammation"[tw] OR "immunothrombosis"[tw] OR "microthrombosis"[tw] OR "microclots"[tw] OR "fibrin amyloid"[tw] OR "platelet activation"[tw]

4 | "Venous Thromboembolism"[Mesh] OR "Pulmonary Embolism"[Mesh] OR "Deep Vein Thrombosis"[Mesh] OR "venous thromboembolism"[tw] OR "VTE"[tw] OR "pulmonary embolism"[tw] OR "PE"[tw] OR "deep vein thrombosis"[tw] OR "DVT"[tw] OR "arterial thrombosis"[tw] OR "cerebral venous thrombosis"[tw]

5 | "Neutrophil Extracellular Traps"[Mesh] OR "Antiphospholipid Syndrome"[Mesh] OR "neutrophil extracellular traps"[tw] OR "NETs"[tw] OR "NETosis"[tw] OR

"antiphospholipid antibodies"[tw] OR "lupus anticoagulant"[tw] OR "anti-cardiolipin antibodies"[tw] OR "anti-β2-glycoprotein I"[tw] OR "von Willebrand Factor"[tw] OR "VWF"[tw] OR "ADAMTS13"[tw] OR "Cytokine Release Syndrome"[Mesh] OR "cytokine storm"[tw]

6 | #2 OR #3 OR #4 OR #5

7 | #1 AND #6

8 | #7 NOT (("animals"[Mesh] NOT "humans"[Mesh]) OR "comment"[Publication Type] OR "editorial"[Publication Type] OR "case report"[Publication Type])

Filters: from inception to September 30, 2025; English language.

Search Strategy Notes:

1. [Mesh]: Denotes a Medical Subject Heading (MeSH term).

2. [tw]: Denotes a text word search (Title/Abstract).

3. Adaptation for Other Databases:

- For Scopus, the search was translated using the same conceptual blocks, using the TITLE-ABS-KEY field and Emtree terms where applicable.

- For Web of Science, the search was translated using the TS (Topic) field, which searches titles, abstracts, author keywords, and Keywords Plus®.

4. Limits: The final PubMed search was limited to human studies and excluded specific publication types (comments, editorials, case reports) as per the pre-registered protocol. These same conceptual limits were applied in the other databases where possible.
